# The effect of oropharyngeal mother’s milk on nutritional outcomes in preterm infants: a randomized controlled trial

**DOI:** 10.1186/s12887-024-04621-5

**Published:** 2024-03-04

**Authors:** Fatemeh Kelich, Mojtaba Qanbari Qalehsari, Ali Zabihi, Seyedeh Roghayeh Jafarian Amiri, Navid Danaee

**Affiliations:** 1 Student Research Committee, Babol University of Medical Sciences, Babol, Iran; 2 Nursing Care Research Center, Health Research Institute, Babol University of Medical Sciences, Babol, Iran; 3 Social Determinants of Health Research Center, Health Research Institute, Babol University of Medical Sciences, Babol, Iran; 4 Nursing Care Research Center, Health Research Institute, Babol University of Medical Sciences, Babol, Iran; 5 Pediatric Research Center, School of Medicine, Associate Professor of Neonatal-Perinatal Medicine, Semnan University of Medical Sciences, Semnan, Iran

**Keywords:** Premature, Infant, Oropharyngeal methods, Breast milk, Nutritional, Outcomes

## Abstract

**Background and objective:**

Oropharyngeal interventions are an accepted method to improve the nutritional performance of premature infants. Considering the countless benefits of breast milk and the few studies on the use of breast milk as an oral-pharyngeal intervention, this study was conducted with the aim of determining the effect of oral-pharyngeal administration of breast milk on nutritional outcomes in premature infants.

**Materials and methods:**

In this clinical trial, 80 premature infants hospitalized in the neonatal intensive care unit of Amir al-Mu’minin Hospital in Semnan (a city in Iran) were randomly assigned to intervention (*n* = 40) and control groups (*n* = 40). Infants in the intervention group were given breast milk, and infants in the control group were given sterile water as a placebo. The data collection tool included demographic and clinical questions checklist, including sex, gestational age, weight, milk administration time, lavage and its amount, vomiting, abdominal distension, and so on. Data analysis was performed using SPSS23.

**Results:**

The mean volume of total milk received by infants (*p* = 0.047) and the mean volume of milk received by mouth (*p* < 0.000) at the time of discharge were higher in the intervention group. Moreover, the time to start enteral nutrition in the intervention group was lower than in the control group (*P* = 0.012). Administering mother’s milk through the oropharyngeal method led to a reduction in infants’ length of stay in the hospital (*P* = 0.022).

**Conclusion:**

Based on the results of the present study, the oropharyngeal administration of breast milk in the first days after the birth of premature infants admitted to the hospital improves the outcomes related to their nutritional status. Therefore, it is suggested that this convenient, safe, and feasible method be used in hospitalized premature infants as soon as possible so that premature infants can benefit from the important advantages of breast milk.

## Introduction

Prematurity of an infant is a major health problem. One of the main challenges of treating a premature infant who is deprived of the food reserves of the placenta and experiences rapid extra uterine growth is nutritional care and proper nutrition for sufficient growth [1]. A universal criterion commonly used for discharge from the neonatal intensive care unit is the infant’s ability to consume milk by mouth, known as full oral feeding [2].

In addition to nutritional benefits, breast milk has biological and probiotic activity, sometimes referred to as “liquid gold” in some studies [3]. Furthermore, this milk contains various substances such as growth factors, important digestive enzymes, immune and anti-inflammatory factors, antioxidants, stem cells, and hormones [4]. Therefore, breastfed infants are usually less exposed to infection, necrotizing enterocolitis, retinopathy of prematurity, chronic lung disease, and bronchopulmonary dysplasia [3, 5].Human milk, produced in the first days after the birth of an infant, is called colostrum, and its secretion continues for 2 to 5 days. Colostrum contains a high amount of immune factors that stimulate the infant’s defense system [6]. The first feeding of an infant with colostrum has many positive outcomes, especially for very premature infants who were not exposed to the growth factors in the amniotic fluid during the last three months [7].Early exposure to this vital extract in premature infants is limited due to maternal reasons. In addition, the feeding of this group of infant is delayed due to clinical instability [8]. Moreover, administering small amounts of milk through the stomach tube may not be practical [3]. As a result, the premature infant is deprived of this valuable liquid, leading to increased sensitivity to various infections and inflammatory conditions [9].

Milk does not come into contact with the mouth through gavage feeding, resulting in the deprivation of the oral cavity and oropharyngeal pouch of the effects of milk [10]. On the other hand, enteral nutrition is also not faultless since premature infants’ condition sometimes notifies the medical staff of a problem called nutritional intolerance. Nutritional tolerance is defined as a condition in which the premature infant digests the received milk, has less than 30% of the previously received lavage, and does not experience vomiting, abdominal distention, or bile secretions. The occurrence of any of these cases can be a sign of necrotizing enterocolitis and nutritional intolerance [1]. Nutritional intolerance is observed in 16–19% of premature infants, which can cause prolonged hospitalization, increased risk of infection, and long-term intravenous feeding [1, 8].

Management of oral nutrition for preterm infants is one of the main aspects of care [11]. One way that can provide the infant the opportunity to access the countless benefits of this precious liquid is to give breast milk by the oropharyngeal method [5]. Oropharyngeal administration of milk is a straightforward method of feeding for premature infants, which has local immunostimulating effects, reduces the risk of sepsis, facilitates enteral feeding, and has other positive results [3, 5, 11, 12]. Therefore, according to the results of the studies conducted on the physiological barriers to the initiation of oral feeding and the importance of initiating oral feeding with breast milk as soon as possible in premature infants and since there are few studies on the methods of facilitating the initiation of oral feeding with breast milk in premature infants hospitalized in the neonatal intensive care unit, this study was conducted with the aim of determining the effect of oropharyngeal administration of breast milk on nutritional outcomes in premature infants.

## Materials and methods

This clinical trial was conducted on 80 premature infants admitted to the Neonatal Intensive Care Unit (NICU) of Amir al-Mu’minin Hospital in Semnan (a city in Iran) between January 2022 and May 2023. In order to calculate the sample size, based on the previous study [13], the effect size: 0.6, the confidence level: 0.95 and the power: 0.8, the sample size in this study was estimated to be 40 sample. The present study was registered in Iran’s clinical trial site under the code IRCT20220509054798N1 on 21.05.2022.

The initial sampling was performed through the convenience method. Afterward, the infants were assigned to groups of 40, A and B, using the random allocation method with blocks of 4. The random allocation list was prepared by the software using the Sealed Envelope website. The randomized list was designed by a statistical researcher assistance and the order of the groups was written on cards based on the randomized list. By the time the researcher declared the infant’s eligibility, the type of intervention was provided to that individual. All researchers, doctors, nurses, and parents were blind to the randomization, and only The researcher in the ward who prepared the placebo syringes and breast milk was aware of it. For each dose of milk or placebo, two prepared syringes were required. Considering the intervention administered every two hours, a total of 24 insulin syringes were required for 24 h. After preparing the syringes, the researcher in the ward completely covered the syringes with a matte cover such as paper or Leucoplast glue to make the contents unrecognizable. Afterward, the name of the infant and the date of collection and packaging were mentioned on each syringe. A separate box for storing syringes in the refrigerator was considered for each infant, and the nurse prescribed the syringes prepared by the researcher, which were covered, to the infants.

The inclusion criteria included infants with a gestational age between 25 and 30 weeks, and the exclusion criteria included congenital abnormalities in the digestive or renal system such as omphalocele, gastroschisis, mother’s addiction, mother’s infection with HIV, and mother’s prohibition to breastfeed. Infants who required a ventilator or developed necrotizing enterocolitis or other disorders prohibiting them from receiving milk during the study were also excluded. The factors that improve nutritional tolerance, including kangaroo care and non-nutritional sacking exercises, were observed equally for all infants participating in the study.

**Fig. 1:**
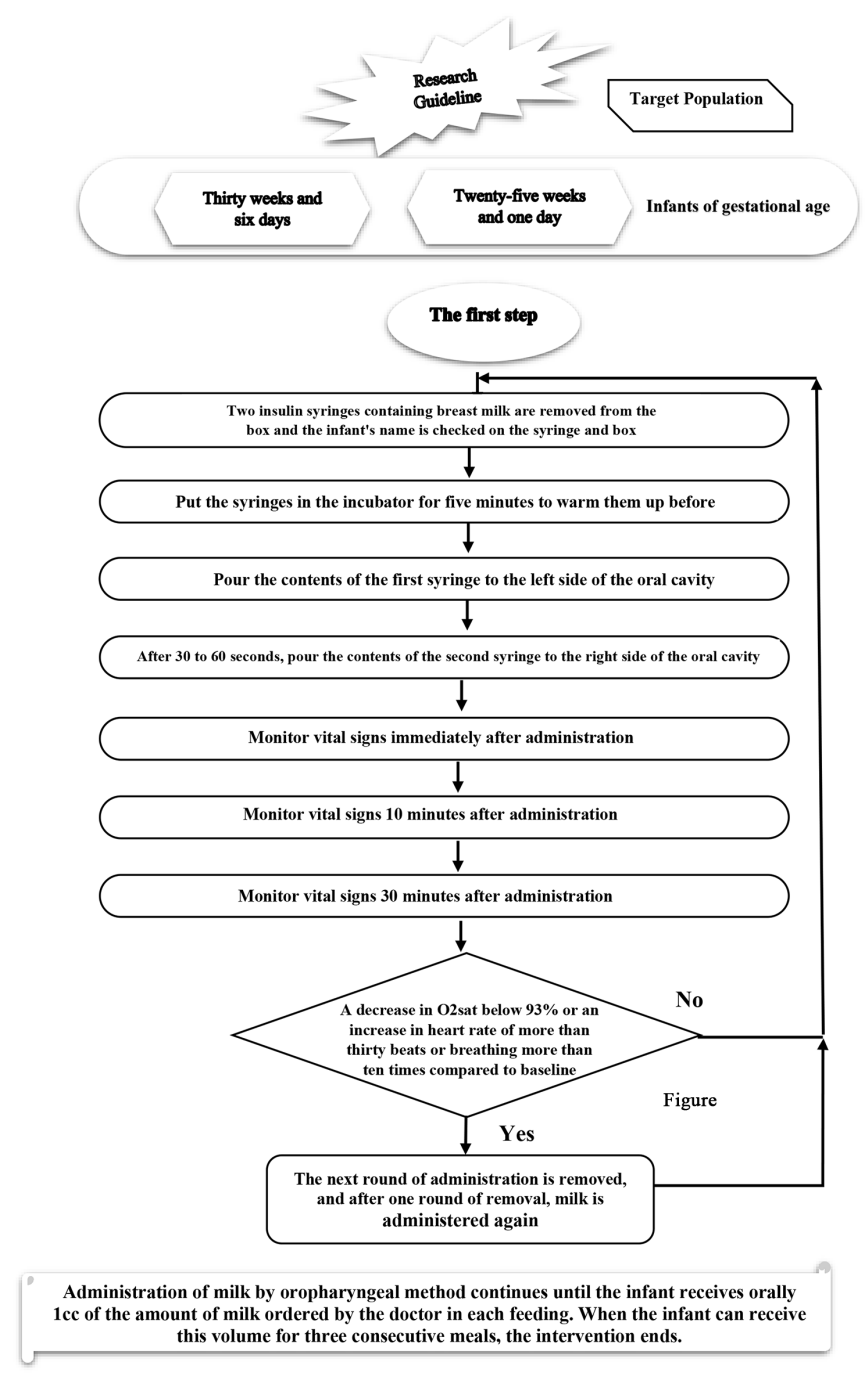
Instructions on how to conduct research project

Data were collected using the demographic and clinical checklist, which included the infant’s sex, gestational age, weight, hourly milk administration time, recording vital signs during, ten minutes, and thirty minutes after administration, lavage and its amount, vomiting and its frequency, and abdominal distention. This checklist was prepared by the researchers and reviewed by 12 experts in the nursing care of the newborn. Moreover, its validity was checked and confirmed. After obtaining permission from the ethics committee with the code (IR.MUBABOL.REC.1400.281), the researcher attended the NICU of the hospital and explained the study procedure to the head nurse and the NICU nurses during a meeting. In addition, the researcher (one of the NICU staff) was present in the ward for a week during the shift handover, explained the procedure to the staff once more, and answered their questions. After the necessary training, the researcher prepared the procedure step by step as a platform and placed it in the nursing station, visible to all the personnel (Fig. 1). In this study, the outcomes related to nutritional status, including the total volume of milk received at the time of discharge, the volume of oral milk received at the time of discharge and the weight at the time of discharge, are the primary outcome and the time to start enteral feeding is the secondary outcome.

After obtaining informed consent, the researcher met the mothers of the studied infants within 24 h after delivery and taught them how to express milk by hand and using an electric breast pump. Sterile containers were given to the mothers of both groups to collect milk. They were also taught to pour the milk into the containers after washing their hands, collect it, and immediately send it to the NICU for storage. If the mother was discharged from the hospital and expressed her milk at home, she was required to pour it into the mentioned container and put it in the freezer and put a date on it, or hand it to the NICU staff in the container surrounded by the ice to keep it cold.

Insulin syringes, made by Teb Iran Company, were used to administer milk. The researcher prepared insulin syringes with 0.1 ccs of breast milk for the intervention group infants and 0.1 ccs of sterile water for the control group infants by following aseptic techniques, including careful hand washing and using sterile syringes in a clean environment. Then she covered the syringe with an opaque sheet in such a way that the contents inside were not visible. The milk of mothers in the control group was stored in the freezer until the start of milking by gavage method. If the amount of milk of mothers in the intervention group was more than needed, their milk was also stored.

The intervention was initiated 48 to 72 h after the birth of each infant. For each meal, two syringes were taken out of the box related to the infant, and the name on the syringe and the box was checked. Syringes were placed in an incubator for five minutes to warm up before administration. After heating the syringes, the content of the first syringe was poured into the left side of the oral cavity, and after 30–60 s, the content of the second syringe was poured into the right side of the oral cavity. Infants in the intervention group received breast milk, and infants in the control group were given sterile water as a placebo, following the same method and protocol. This procedure was repeated every two hours. The intervention was continued after starting enteral feeding, which included milk gavage until the infant received one cc of the milk ordered by the doctor per meal orally. When the infant was able to receive this volume for three consecutive meals, the intervention ended for them.

Vital signs, including heart rate, breathing, and oxygen saturation of the infant’s blood, were recorded at the time of administration and ten and thirty minutes after administration. If the blood oxygen saturation dropped below 93%, the heart rate increased to thirty beats above the baseline, or breathing occurred ten times more than the baseline, the subsequent milk administration was removed and continued according to the schedule after one round removed.

The data were entered into SPSS23 software after being collected. Independent sample t-test were used to measure and compare quantitative variables between the two groups. A significance level was considered less than 0.05.

## Results

**Fig. 2:**
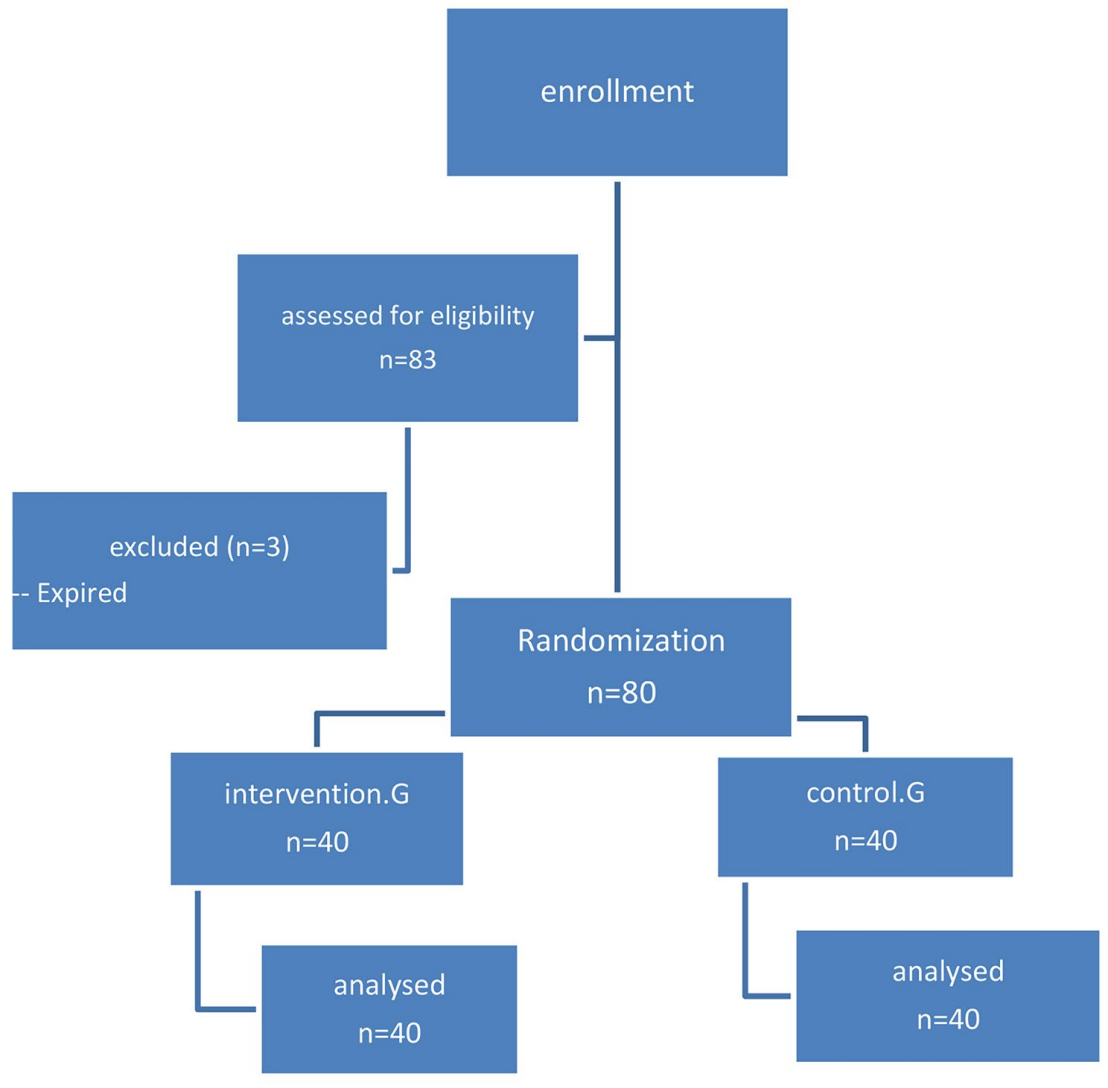
Selection of the study participants

In this study, 80 premature infants with a gestational age of 25 to 30 weeks were examined in two groups, namely intervention and control (Fig. 2). The frequency distribution and percentage of demographic characteristics of infants in intervention and control groups are shown in Table 1. In this study, 12.05% of the mothers experienced COVID-19; however, none of the infants were affected by this disease.

**Table 1:** Frequency distribution and percentage of individual characteristics of the studied infants

Variables		Intervention Group N (%)	Control Group N (%)
Sex	boy	20 (50)	21 (51.22)
	girl	20 (50)	19 (48.72)
Weight (gr)	< 1000	10 (25)	9 (22.5)
	1000–1500	20 (50)	27 (67.5)
	< 1500	10 (25)	4 (10)
Gestational age (week)	25–26	5 (12.5)	6 (15)
	27–28	12 (30)	9 (22.5)
	29–30	23 (57.5)	25 (62.5)

The study groups were not significantly different from each other in terms of variables related to infant care. Table 2 shows the data related to care variables.

**Table 2:** Mean and deviation of some variables in infants of intervention and control groups

Variables	Groups	Mean ± deviation	*P*-value
lavage (cc)	Intervention (*n* = 40)	16.57 ± 12.72	0.07
	Control (*n* = 40)	21.64 ± 12.46	
Kangaroo mother care(hours)	Intervention (*n* = 40)	26.46 ± 11.20	0.43
	Control (*n* = 40)	28.25 ± 9.23	
speaking (minutes)	Intervention (*n* = 40)	224.53 ± 98.27	0.42
	Control (*n* = 40)	255.28 ± 223.62	
Meeting (minutes)	Intervention (*n* = 40)	2141.75 ± 756.53	0.53
	Control (*n* = 40)	2241.20 ± 656.77	
Non-nutritional sucking exercise (minutes)	Intervention (*n* = 40)	79.05 ± 38.89	0.16
	Control (*n* = 40)	90.87 ± 36.31	

A comparison of the study groups in terms of nutritional outcomes showed that in the intervention group, infants’ condition in terms of total milk received at discharge, oral milk at discharge, hospitalization period, and the start of enteral feeding was significantly better than the control group (Table 3).

**Table 3:** Nutritional outcomes of the mother’s milk administration based on the oropharyngeal method in premature infants

Variables	Groups	Mean ± deviation	Eta (ES)	MD	Confidence interval (95%)	lower-higher	*P*-value
Total milk received at discharge (cc)	Intervention	27.87 ± 2.93	0.016	1.74	0.656, 2.824	24–36	0.047
	Control	26.13 ± 1.81				22–29	
Oral milk at discharge (cc)	Intervention	3.80 ± 1.42	0.004	1.88	1.331, 2.428	1–16	< 0.001
	Control	1.92 ± 1.01				1–4	
Weight at discharge (gr)	Intervention	1600.48 ± 199.36	0.028	76.63	-1.589, 154.849	780–2030	0.116
	Control	1523.85 ± 148.33				1100–1790	
Hospitalization period (days)	Intervention	30.80 ± 11.58	0.019	6.33	-11.639, 0.966	10–50	0.022
	Control	37.13 ± 12.50				17–60	
Start of enteral feeding (days)	Intervention	4.61 ± 2.39	-	1.32	-2.336, -0.304	2–10	0.012
	Control	5.93 ± 2.17				3–10	
lavage (cc)	Intervention	16.57 ± 12.46	-	5.07	-10.674, -0.534	0–53	0.074
	Control	21.64 ± 12.72				0–63	

MD (95% CI): mean difference and its 95% confidence interval.

## Discussion

In this study, the volume of milk received oropharyngeal at the time of discharge in premature infants of the intervention group was significantly higher than in the control group, indicating the positive effect of oropharyngeal milk administration on nutritional outcomes in premature infants. Snyder et al.’s study likewise showed that the administration of colostrum by the oropharyngeal method in premature infants had positive results in the initiation of oral feeding and its tolerance [5]. In the clinical trial conducted by Rodriguez et al., oral oropharyngeal administration of breast milk in preterm infants in the intervention group compared to the control group that received a placebo led to earlier initiation of complete oral feeding and enteral feeding [14]. Another similar study indicated that the oral administration of breast milk in premature infants accelerated the onset of enteral feeding [15]. In the study by Le et al., it was shown that oral stimulation of premature infants with breast milk, compared to simple oral stimulation, significantly shortened the time to start oral feeding and resulted in an earlier transition from oral feeding to complete oral feeding [16]. Oral intervention improves swallowing performance by improving sucking performance and can lead to improved nutritional results [17]. The better nutritional results of oral intervention with breast milk compared to sterile water are probably due to the stimulation of nutritional enzyme activity. Studies show that premature infants have special nutritional support needs, and breast milk is recommended as the exclusive diet for infants because it has countless nutritional and safety benefits that should be given special attention.

The results of the present study showed that the initiation of enteral feeding in preterm infants given breast milk via the oropharyngeal route was earlier than in preterm infants of the control group. In the meta-analysis by Kumar et al., which examined oropharyngeal administration of breast milk in premature infants, it was shown that full enteral feeding was started 1.75 days earlier in the intervention group. Considering that many premature infants often cannot be fed through the intestines and take benefits of breast milk advantages, especially colostrum, and due to the low cost and minimal risk of injury, the oropharyngeal administration of breast milk in the first days of birth is emphasized [18]. Abd-Elgawad et al. showed that oropharyngeal administration of breast milk before starting gavage in preterm infants led to earlier initiation of enteral feeding [10]. Recent evidence shows that the intervention of oropharyngeal administration of breast milk has numerous benefits, including early access to full enteral nutrition, increased oral feeding skills, improved growth, and improved breastfeeding outcomes for preterm infants [19]. The earlier initiation of enteral feeding in the group that used breast milk was probably due to the presence of hormones, enzymes, fatty acids, and other biofactors in breast milk that accelerates intestinal maturation and movement in infants [20]. Achieving complete enteral nutrition faster and without adverse effects is essential for premature infants’ proper growth and development.

In this study, one of the important nutritional results of the oropharyngeal administration of breast milk was the examination of the studied premature infants’ weight at the time of discharge. The difference in the infants’ weight at the time of discharge was not significant between the two groups; however, by examining other results, such as the duration of hospitalization in the infants of the two study groups, it can be concluded that the infants in the intervention group reached the appropriate weight for discharge about a week earlier than the infants in the control group. In fact, the weight gain of infants in the intervention group was better than that of the control group. In other words, the intervention of oropharyngeal administration of breast milk can be more effective in gaining weight in the intervention group than the group that received sterile water. In the study by Kumar et al., the oropharyngeal administration of breast milk was significantly effective in infants’ weight gain [19]. Seigel et al. reported that the initiation of oropharyngeal administration of breast milk in the first two days after birth in premature infants was safe and accompanied by beneficial nutritional results, including better weight gain [21]. However, in the study by Snyder et al., the changes in the weight of infants in the group of oropharyngeal administration of breast milk were not significant compared to the control group [5]. In the present study, the length of hospital stay in infants of the oropharyngeal administration of the breast milk group was shorter than in the control group. The results of the present study regarding faster access to enteral nutrition and appropriate weight gain for discharge are consistent with the study by Garfalo [22], Abd-Elgawad [10], and Moreno [23]. It is important to discharge the infant from the hospital even one day earlier due to reducing the risk of hospital infection and treatment costs. In addition, it will psychologically help the infants’ families and it is also effective in premature infants’ neuropsychomotor development [10].

The comparison of the lavage amount in the group with breast milk administration compared to the placebo group, the results of our study showed a decrease in the amount of lavage; however, this change was not significant. This decrease was probably due to the enzymes present in breast milk, which are influential in the maturation and peristaltic movements of the infants’ intestines and were more beneficial to intestinal function in the intervention group. As a result, the volume of the remaining digestive tract (lavage) in the intervention group was lower than in the control group. In the review of other studies, no report was found explicitly examining lavage and gavage variables. According to Tao et al.’s study, the oropharyngeal administration of breast milk reduces the time to complete enteral feeding, improves nutritional tolerance in premature infants, and has positive effects in reducing necrotizing enterocolitis (NEC), late-onset sepsis (LOS), death and hospital stay length in preterm infants [12].

One of the strengths of our study is that this study was conducted for the first time in Iran. One of the limitations of the study was the constant recommendation to mothers to perform Kangaroo care and non-nutritional sucking practice, which required mothers’ cooperation and was sometimes unfeasible due to the number of problems. Another limitation of the study was the lack of timely secretion of breast milk, which was not secreted until 72 h after the mother gave birth. Moreover, some mothers in this study lived far from the research environment. As a result, we sometimes faced problems in performing some maternal care related to this research.

## Conclusion

According to the results of the present study, the administration of breast milk by the oropharyngeal method in the first days of the birth of hospitalized premature infants leads to improved nutritional outcomes in them, including an increase in the total volume of milk received, an increase in the oral intake of breast milk at discharge, an earlier start of enteral feeding, and shorter length of the hospital stay. Therefore, considering the safety, convenience, practicality, and cost-effectiveness of the oropharyngeal administration of breast milk, it is suggested that this method be used in hospitalized premature infants as soon as possible and considered a priority so that premature infants can take the benefit of the breast milk advantages. In addition, for better quality scientific evidence, more studies with standard protocols for oropharyngeal administration of breast milk and a larger sample size are recommended.

## Data Availability

The datasets generated and/or analyzed during the current study are not publicly available due [individual privacy could be compromised] but are available from the corresponding author on reasonable request.

## Abbreviations

NICU: Neonatal Intensive Care Unit
HIV: Human immunodeficiency virus
NEC: Necrotizing enterocolitis
LOS: Late-onset sepsis
